# Biofilm-Resistant Nanocoatings Based on ZnO Nanoparticles and Linalool

**DOI:** 10.3390/nano11102564

**Published:** 2021-09-29

**Authors:** Vera Alexandra Spirescu, Raluca Șuhan, Adelina-Gabriela Niculescu, Valentina Grumezescu, Irina Negut, Alina Maria Holban, Ovidiu-Cristian Oprea, Alexandra Cătălina Bîrcă, Bogdan Ștefan Vasile, Alexandru Mihai Grumezescu, Ludovic Everard Bejenaru, George Dan Mogoşanu, Cornelia Bejenaru, Paul Cătălin Balaure, Ecaterina Andronescu, Laurenţiu Mogoantă

**Affiliations:** 1 Department of Science and Engineering of Oxide Materials and Nanomaterials, University Politehnica of Bucharest, 011061 Bucharest, Romania; veraspirescu@gmail.com (V.A.S.); suhanraluca@yahoo.com (R.Ș.); niculescu.adelina19@gmail.com (A.-G.N.); ada_birca@yahoo.com (A.C.B.); bogdan.vasile@upb.ro (B.Ș.V.); grumezescu@yahoo.com (A.M.G.); ecaterina.andronescu@upb.ro (E.A.); 2 Lasers Department, National Institute for Lasers, Plasma and Radiation Physics, 077125 Magurele, Romania; valentina.grumezescu@inflpr.ro (V.G.); negut.irina@inflpr.ro (I.N.); 3 Department of Microbiology and Immunology, Faculty of Biology, University of Bucharest, 077206 Bucharest, Romania; alina_m_h@yahoo.com; 4 Department of Inorganic Chemistry, Physical Chemistry and Electrochemistry, Faculty of Applied Chemistry and Materials Science, Politehnica University of Bucharest, 011061 Bucharest, Romania; ovidiu73@yahoo.com; 5 Research Institute of the University of Bucharest—ICUB, University of Bucharest, 050657 Bucharest, Romania; 6 Academy of Romanian Scientists, Ilfov No. 3, 50044 Bucharest, Romania; 7 Department of Pharmacognosy & Phytotherapy, Faculty of Pharmacy, University of Medicine and Pharmacy of Craiova, 200349 Craiova, Romania; ludovic.bejenaru@umfcv.ro (L.E.B.); george.mogosanu@umfcv.ro (G.D.M.); 8 Department of Pharmaceutical Botany, Faculty of Pharmacy, University of Medicine and Pharmacy of Craiova, 200349 Craiova, Romania; cornelia.bejenaru@umfcv.ro; 9 Department of Organic Chemistry, Faculty of Applied Chemistry and Materials Science, Politehnica University of Bucharest, 011061 Bucharest, Romania; 10 Research Center for Microscopic Morphology and Immunology, University of Medicine and Pharmacy of Craiova, 200349 Craiova, Romania; editor@rjme.ro

**Keywords:** zinc oxide nanoparticles, linalool, laser processing, matrix-assisted pulsed laser evaporation, antimicrobial resistance, antimicrobial coatings, antibiofilm activity

## Abstract

Biofilms represent an increasing challenge in the medical practice worldwide, imposing a serious threat to public health. As bacterial strains have developed antibiotic resistance, researcher’s attention has been extensively focused on developing more efficient antimicrobial strategies. In this context, the present study reports the synthesis, physicochemical characterization, ex vivo biodistribution, and in vitro evaluation of the capacity of nanostructured surfaces based on zinc oxide (ZnO) and biologically active molecules to modulate clinically relevant microbial biofilms. ZnO nanoparticles (NPs) were synthesized through a co-precipitation method without thermal treatment. The matrix-assisted pulsed laser evaporation (MAPLE) was applied for preparing nanostructured coatings based on ZnO NPs surface modified with linalool that were further characterized by X-ray diffraction (XRD), thermogravimetric analysis with differential scanning calorimetry (TGA-DSC), scanning electron microscopy (SEM), transmission electron microscopy with selected area electron diffraction (TEM-SAED), Fourier-transform infrared spectroscopy (FT-IR), and infrared microscopy (IRM). Histological analyses carried out at 7 days and 14 days after the intraperitoneal administration of linalool modified ZnO NPs revealed the absence of the latter from the brain, kidney, liver, lung, myocardium, and pancreas. Through in vitro assays on prokaryotic cells, it was proven that ZnO coatings hinder microbial biofilm formation of both Gram-positive and Gram-negative bacteria strains.

## 1. Introduction

A biofilm is a community of bacterial cells embedded in a self-derived extracellular polymeric substance (EPS) matrix attached to a biotic or abiotic surface. In the biofilm, bacteria exhibit a series of striking features differentiating them from their planktonic freely suspended counterparts. Among these are the reduced growth rate and the altered gene expression. The social biofilm lifestyle relies on intricate intercellular molecular communication mechanisms that include bacterial cells and pathogen–host chemical signaling systems, enabling the pathogens to resist different stress factors, including host defense mechanisms and antibiotics [1,2,3,4,5,6]. Bacteria growing in a biofilm are 100 to 1000 times less sensitive to antimicrobials than free-floating bacteria, raising a serious threat to public health [7]. A study carried out recently by the European Centre for Disease Prevention, and Control (ECDC) estimated that 1 in 15 hospital patients acquire at least one healthcare-associated infection (HAI) in European Union (EU) and European Economic Area (EEA) countries daily. Hence, a total of 4.5 million HAIs were estimated to occur each year in European hospitals and an additional 4.4 million HAIs in long-term care facilities [8]. According to the National Health Institute (NHI), in the USA, recalcitrant biofilms are responsible for 65% of microbial diseases and 80% of chronic infections [9]. In this crisis context, researchers are urged to develop new alternatives to classical antibiotic therapy which can effectively fight against multidrug resistance (MDR) pathogenic biofilm infections.

The cutting-edge advances in the field of nanostructures and nanotechnology found a broad field of applications in nanomedicine and pharmaceutical treatments, including antibacterial therapy [10,11,12,13]. Metal oxide nanoparticles are extensively studied as valuable alternatives to antibiotics due to a series of interesting characteristics such as proved antimicrobial activity, biocompatibility, lack of toxicity, and stability [14,15,16,17,18,19]. Among these, ZnO has been recognized as a safe food additive by the U.S. Food and Drug Administration (FDA) [20,21]. The antimicrobial activity of ZnO NPs is even greater than that of the bulk material due to their small dimensions, which are size-correlated to the biological systems they interact with, facilitating intracellular uptake, as well as to their high surface to volume ratio associated with high surface energy [20,22,23,24,25,26,27,28]. ZnO is a *n*-type semiconductor, and, after cellular internalization, ZnO NPs generate harmful reactive oxygen species (ROS) through a series of oxidation-reduction reactions initiated either photochemically or by the surface defects of the nanomaterial. Further, the produced ROS induce increased oxidative stress, damaging the bacterial cell and its components [29,30,31,32].

Essential oils (EOs) are a group of natural products with great therapeutic potential due to a plethora of pharmaceutically useful properties, especially their antimicrobial, antiseptic, analgesic, anti-inflammatory, and antioxidant activities [33,34,35]. Particularly, the hydrophobic nature of EOs enables them to cross bacteria cell membranes and destroy cell-wall structures [36,37,38].

Because many HAIs produced by antibiotic-recalcitrant biofilms are related to implantable and indwelling medical devices, causing a substantial increase in therapeutical costs and even life-threatening consequences [4,6,31,32,33,34,35], there is an imperious need to protect the surface of such devices with biofilm-resistant coatings.

The matrix-assisted pulsed laser evaporation (MAPLE) method is used to deposit hybrid nanostructures on target surfaces [39]. MAPLE has attracted interest in engineering nanostructured antimicrobial coatings due to a series of advantages, including enhanced substrate adhesion and preservation of the transferred material’s chemical integrity and physicochemical properties [40,41,42,43].

Herein, we report the MAPLE fabrication of novel nanostructured coatings based on ZnO NPs surface modified with linalool (one of the main components of the lavender essential oil). The synthesized nanocoatings were fully characterized by physical methods: X-ray diffraction (XRD), thermogravimetric analysis with differential scanning calorimetry (TGA-DSC), scanning electron microscopy (SEM), transmission electron microscopy with selected area electron diffraction (TEM-SAED), Fourier-transform infrared spectroscopy (FT-IR), and infrared microscopy (IRM). The antibacterial and antibiofilm activity was tested against relevant microorganisms responsible for HAIs and biofilm infections, such as Gram-positive *Staphylococcus aureus* (*S*. *aureus*), and *Enterococcus faecalis* (*E*. *faecalis*) and Gram-negative *Escherichia coli (E. coli)*, and *Pseudomonas aeruginosa* (*P*. *aeruginosa*) model strains.

## 2. Materials and Methods

### 2.1. Materials

All chemicals used to synthesize nanostructured materials, namely zinc nitrate hexahydrate 98% Zn(NO_3_)_2_·6H_2_O, sodium hydroxide 97% NaOH, methanol CH_3_OH, dimethylsulfoxide (DMSO), linalool, and polyvinylpyrrolidone (PVP) were purchased from Sigma Aldrich (Merck Group, Darmstadt, Germany) and used without any further purification.

### 2.2. Synthesis Methods

#### 2.2.1. ZnO Nanoparticles’ Synthesis

For the synthesis of ZnO NPs, we used the co-precipitation method without thermal treatment [44]. This method involves two solutions. The first solution contained the Zn precursor and was prepared by dissolving 3 g of zinc nitrate hexahydrate in 100 mL methyl alcohol under stirring until proper homogenization. The second solution is composed of 3 g NaOH in 100 mL of deionized water and acts as the precipitation reagent. A control sample of ZnO nanoparticles was prepared by dropwise addition of the alkaline precipitation reagent solution to the Zn precursor solution and stirring. The initial transparent solution gradually turns milky white. The product was collected by centrifugation, and the precipitate was further washed with deionized water and air-dried.

#### 2.2.2. Synthesis of ZnO Nanoparticles Surface Modified with Linalool (ZnO@LiN)

ZnO NPs surface-functionalized with linalool were prepared following the above-described procedure. The only difference is that 500 µL of the functionalizing agent (linalool) was added in the precipitation alkaline reagent solution.

#### 2.2.3. Polyvinylpyrrolidone (PVP)-Based Coatings Synthesis

Before surface modification by MAPLE processing, all substrates (silica slides and glass) were subjected to a triple cleaning treatment in the ultrasonic bath with acetone, ethanol, and deionized water for 20 min.

A KrF* excimer laser source (λ = 248 nm, τFWHM = 25 ns), model COMPexPro 205 Lambda Physics from Coherent, was employed for obtained coatings. The MAPLE deposition targets were prepared by suspending the ZnO@LiN previously blended with PVP in DMSO to obtain a 3% (*w*/*w*) suspension. For solid target preparation, suspensions of PVP/ZnO@LiN particles were frozen at liquid nitrogen temperature. During MAPLE processing, the laser spot area was 34 mm^2^ and experimental parameters were maintained constant, including substrate temperature and background pressure (room temperature and 0.1 Pa, respectively), target to substrate distance (5 cm), laser repetition frequency (15 Hz), number of applied laser pulses (60,000). The MAPLE coatings were obtained by irradiating the frozen targets at different laser fluences, namely 200, 300, and 400 mJ/cm^2^.

### 2.3. Physicochemical Characterization

The crystallinity of the ZnO NPs was investigated by XRD analysis carried out on a PANalytical Empyrean diffractometer (Almelo, Netherlands), using CuK α radiation (λ = 1.540598 A), equipped with a 2 × Ge (2 2 0) monochromator for Cu and a PICcel3D detector. Bragg-Brentano parafocusing geometry was used with the X-ray tube fixed while the sample rotates at θ/min and the detector always at 2θ/min (θ:2θ scan). The XRD analysis was accomplished in the Bragg diffraction angle range between 10 and 80° using step scan mode with step sizes of 0.04° and an acquisition time per scanning step of 3 s. The XRD spectra were collected on powders obtained by grinding of the genuine nanomaterial samples.

TGA-DSC analyses were performed using Shimadzu DTG-TA-50H equipment (Carlsbad, CA, USA). Data were collected in the temperature range between 30 and 900 °C at a heating rate of 10 °C/min under an air atmosphere.

Characterization of the surface morphological details of the deposited nanocoatings was accomplished by SEM with an FEI (Hillsboro, OR, USA) electron microscope, using the secondary low energy emission (30 keV) from the sample for high-resolution imaging of the sample’s surface.

TEM was used to reveal the inner structural and morphological characteristics of the NPs. The TEM specimens were prepared as follows. A small quantity of sample powder was dispersed in pure ethanol and subjected to ultrasonic cleaning for 15 min. Next, the sample was placed on a carbon-coated copper grid and left to dry at room temperature. TEM micrographs were recorded using a Tecnai^TM^ G2 F30 S-TWIN instrument equipped with a selected area electron diffraction (SAED) accessory (Hillsboro, OR, USA), and operated at 300 kV. The guaranteed TEM point resolution and a line resolution are 2 Å and 1.02 Å, respectively.

To confirm the preservation of the structural integrity of the deposited coatings of bare and surface-modified ZnO NPs after laser processing at various fluences, we used the infrared microscopy (IRM) technique. IR maps acquisition was performed with a Nicolet iN10 MX FT-IR Microscope equipped with a liquid nitrogen-cooled mercury cadmium telluride (MCT) detector. The spectral collection was obtained operating in reflection mode in the wavenumber range from 4000 to 600 cm^−1^ with a spectral resolution of 4 cm^−1^. The collected spectra (32 scans per spectrum) were overlapped, and absorbance maps were created based on the second derivative of the spectral data using Ominc Picta Version 8.0 (Thermo Fischer Scientific Company, Waltham, MA, USA) software.

### 2.4. Antimicrobial Evaluation

The bacterial strains of *Escherichia coli* (ATCC ^®^ 25922), *Pseudomonas aeruginosa* (ATCC ^®^ 27853), *Staphylococcus aureus* (ATCC ^®^ 25923), and *Enterococcus faecalis* (ATCC ^®^ 29212) were obtained from the American Type Cell Collection (ATCC, Manassas, VA, USA).

To establish the minimum inhibitory concentration (MIC) of the utilized nanoparticles, a quantitative method based on binary serial microdilutions in a liquid medium distributed in 96-well plates was used. In the first well of each row was added an amount of sample corresponding to a concentration of 2000 µg/mL. Using a micropipette, binary dilutions were performed by a final concentration of 0.1 µg/mL). Next, in each well were added 15 µL of 0.5 McFarland density microbial suspensions. The as-prepared plates were incubated for 24 h at 37 °C. After incubation, the MIC value for each sample was determined by visual examination as the lowest concentration at which no microbial growth was observed (lack of turbidity). The value was confirmed using a spectrophotometer by reading the absorbance of microbial culture at 620 nm.

To evaluate the ability of the nanostructured surfaces to interfere with biofilm formation, all samples were first sterilized by UV exposure for 20 min on each side. Each fragment of sterile material was then individually placed in a well of a 6-well plate, followed by the addition of 2 mL of nutritive broth and subsequent inoculation of 50 µL of bacterial suspensions (0.5 McFarland suspension (1.5 × 10^8^ CFU (colony forming units)/mL prepared in phosphate-buffered saline, PBS). The as-prepared 6-well plates were incubated at 37 °C for 24 h. After incubation, the samples were washed with PBS, and the culture medium was changed with a fresh one to ensure microbial biofilm development. After 24, 48, and 72 h of incubation, the specimens were gently washed with PBS and transferred to 1 mL PBS-containing sterile tubes. The tubes were vigorously vortexed for 30 s to detach the biofilm cells. The obtained cell suspensions were diluted and seeded on plates with solidified culture medium in order to obtain and quantify the number of colony-forming units (CFU/mL). All experiments were done in triplicate and repeated on 3 different occasions.

### 2.5. In Vivo Biocompatibility and Biodistribution of ZnO NPs

The experimental protocol was applied according to the European Council Directive No. 86/609 (24 November 1986), the European Convention on the Protection of Vertebrate Animals used for Experimental and Other Scientific Purposes (2 December 2005) and the Romanian Parliament Law No. 43 (11 April 2014) on the Protection of Animals used for Scientific Purposes. The study was approved by the Ethics Committee of the University of Medicine and Pharmacy of Craiova, Romania (Approval Certificate No. 118/27 May 2015).

For histological analysis, three months old BALB/c mice were used for ZnO NPs samples and reference. A total of 12 mice were used. Throughout the experiment, the mice were kept in standard water and food conditions (ad libitum). In the groin area, the mice were intraperitoneally inoculated, with 200 µL of 1 mg/mL dispersion of ZnO NPs previously sterilized by UV irradiation for 30 min in sterile saline. Reference mice were intraperitoneally inoculated, in the groin area, with 200 µL of sterile saline. At 7 days and 14 days after the beginning of the experiment, the animals were euthanized, under general anesthesia (Ketamine/Xylazine mixture), for the sampling of internal organs (brain, kidney, liver, lung, myocardium, pancreas, spleen). The biological material was washed directly after the sampling in phosphate-buffered saline (PBS) to remove blood. Then, the internal organs were fixed in 10% neutral buffered formalin, for 72 h, at room temperature and processed for routinely histological paraffin-embedding technique [24,45].

For the histological study of ZnO NPs, 4-µm thick serial sections were cut on a MICROM HM355s rotary microtome (MICROM International GmbH, Walldorf, Germany) equipped with a waterfall-based section transfer system (STS, MICROM). The cross-sections were placed on histological slides treated with poly-L-lysine (Sigma-Aldrich, Munich, Germany). After Hematoxylin–Eosin (HE) classical staining, cross-sections were evaluated and photographed using a Nikon Eclipse 55i light microscope equipped with a Nikon DS–Fi1 CCD high-definition video camera (Nikon Instruments, Apidrag, Romania). Images were captured, stored, and analyzed using Image-Pro Plus 7 AMS software (Media Cybernetics Inc., Marlow, Buckinghamshire, UK).

## 3. Results

### 3.1. Characterization of ZnO and ZnO@LiN Nanopowders

**Figure 1:**
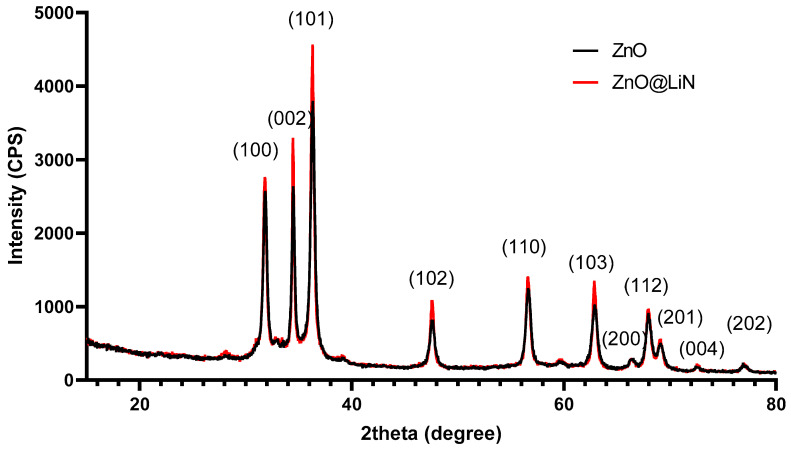
Diffractogram of ZnO NPs and ZnO@LiN NPs.

XRD patterns of prepared bare and surface-modified ZnO nanoparticles are plotted in Figure 1, respectively. The sharp diffraction peaks appearing at specific 2θ diffraction angles correspond to the diffraction planes (100), (002), (101), (102), (110), (103), (200), (112), (201), (004), and (202) which are characteristic for the hexagonal wurtzite phase of ZnO, JCPDS card No: 5–0664 [46], confirming the high purity of the synthesized NPs. The strongest peak in all diffractograms corresponds to the diffraction plane (101). Surface functionalization produces no changes in peak intensities, meaning that the crystalline structure of the nanopowders remains intact.

**Figure 2:**
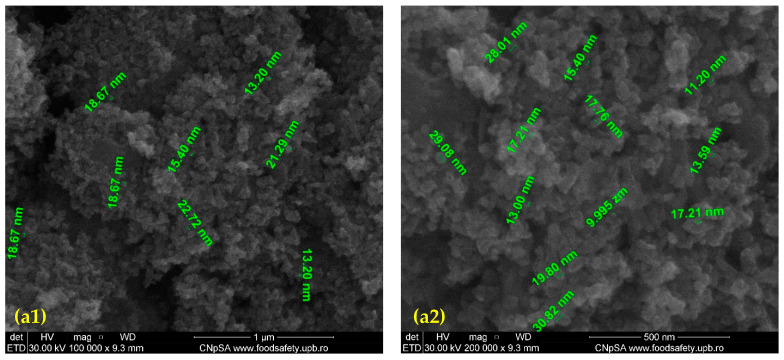

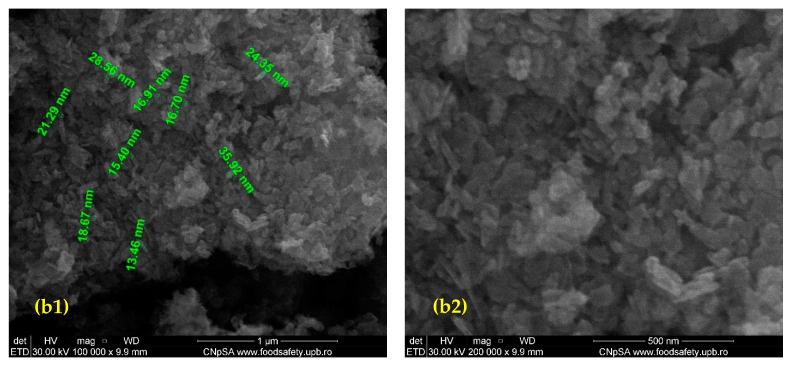
SEM image of bare ZnO NPs (**a1**,**a2**) and ZnO@LiN NPs (**b1**,**b2**).

SEM images of the prepared nanostructured ZnO particles are plotted in Figure 2. At high magnification, it can be observed that the nanoparticle dimensions range from 13 to 20 nm with relatively uniform size distribution and irregular shape. Agglomeration tendency is reduced because only a few agglomerates could be observed. No significant structural changes compared to the bare nanoparticles are visible, revealing that surface functionalization does not affect particles morphology.

**Figure 3:**
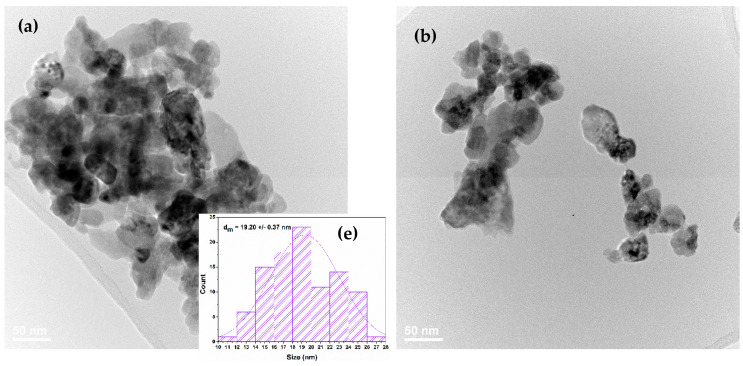

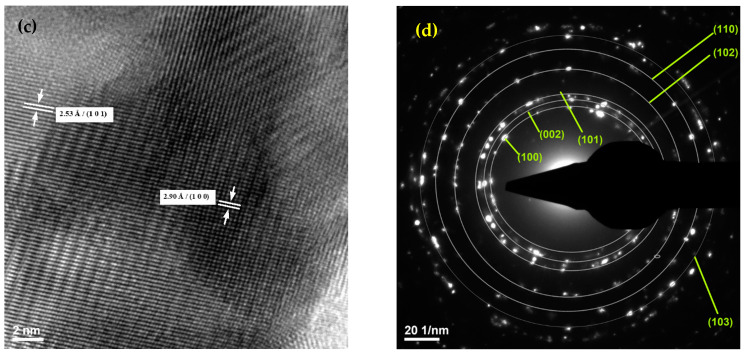
(**a**,**b**) TEM images; (**c**) HRTEM image; (**d**) SAED pattern of ZnO@LiN nanoparticles; (**e**) histogram of particle size-distribution.

TEM and SAED analyses were used to determine the size, aspect, growth direction, and nature of the formed crystalline ZnO phases. TEM micrographs (a) and (b) in Figure 3 show the irregular polyhedron shapes of ZnO nanoparticles with uniform size distribution and a slight agglomeration tendency. The high-resolution transmission electron microscopy (HR-TEM) image in Figure 3c reveals the ordered growth direction of the crystalline planes of the ZnO wurtzite lattice, while the SAED pattern of the concentric diffraction rings visible in Figure 3d are in excellent agreement with the results of the XRD analysis confirming the presence of the same diffraction planes and the polycrystalline nature of the samples [47].

The IR spectra of the prepared bare and surface-modified ZnO NPs (IR spectra not shown). The first absorption band is recorded at 834 cm^−1^, corresponding to Zn-OH bonds, while the second absorption band is seen at 398 cm^−1^, being attributed to Zn-O bonds. The strongest absorption peak is between 400 and 600 cm^−1^ for all samples, highlighting the preponderant ZnO composition. No absorption bands were registered for linalool because their concentration is below the detection limit of the apparatus. In this context, TGA-DSC analysis was used to highlight the amount of the organic compounds that interact with ZnO NPs.

**Figure 4:**
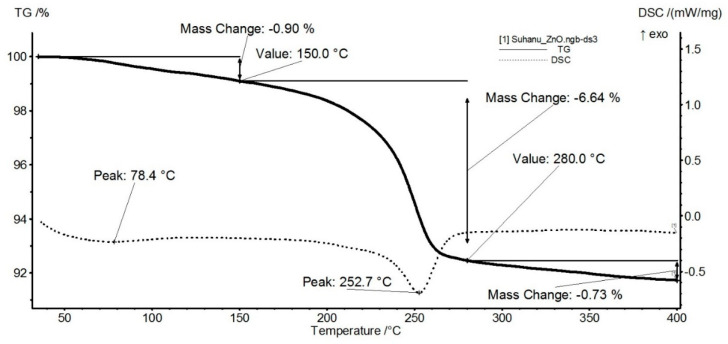
Thermogravimetric analysis of ZnO.

The thermal analysis for the ZnO sample (Figure 4) indicates the presence of three independent mass loss processes. The first mass loss between RT-150 °C represents 0.90% and is due to eliminating residual solvent molecules. The second process, with a mass loss of 6.64%, represents the main decomposition step. It is accompanied by an endothermic effect with a minimum of 252.7 °C. The process can be attributed to the decomposition of some zinc nitrate impurities. The mass loss continues slowly after 280 °C, without noticeable effects on the DSC curve, up to 400 °C 0.73% mass loss being recorded.

**Figure 5:**
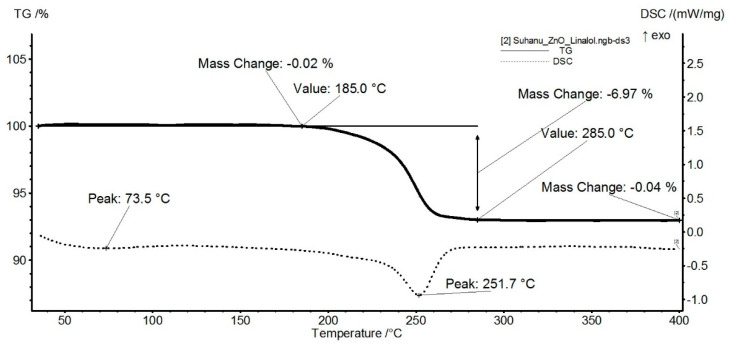
Thermogravimetric analysis of ZnO@LiN.

The thermal analysis for the ZnO@LiN sample (Figure 5) indicates the presence of a single mass loss process. The sample is stable up to 185 °C, with negligible mass loss (0.02%). This indicates that during the loading process, the previous solvent traces were removed. The mass loss of 6.97% is recorded between 185 and 285 °C. The process is accompanied by an endothermic effect, with the minimum at 251.7 °C. The linalool molecules loaded on the surface of ZnO NPs are eliminated in this step (linalool b.p. = 199 °C). By comparing the mass loss values for this interval for both samples (simple ZnO and linalool loaded ZnO), we estimate that the linalool quantity loaded on ZnO is ~0.78%.

### 3.2. Characterization of PVP/ZnO@LiN Coatings

After MAPLE deposition, IR analysis was employed to acquire both spatial and spectral information by comparing the IR data of the dropcast to thin coatings obtained at different laser fluences

**Figure 6:**
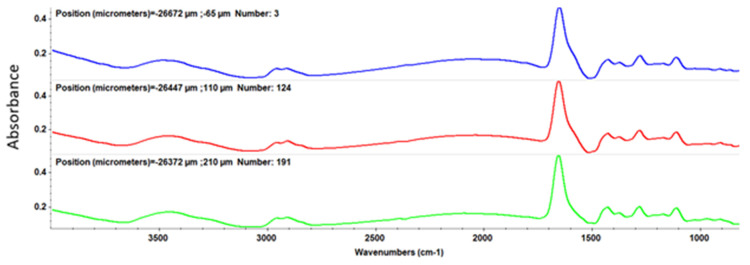
IR spectra of PVP/ZnO@LiN dropcast.

From the IR spectrum of the dropcast (Figure 6), important peaks are observed at 1646.23 cm^−1^ and 3466 cm^−1^, being characteristic of the C=O (OH) bond.

**Figure 7:**
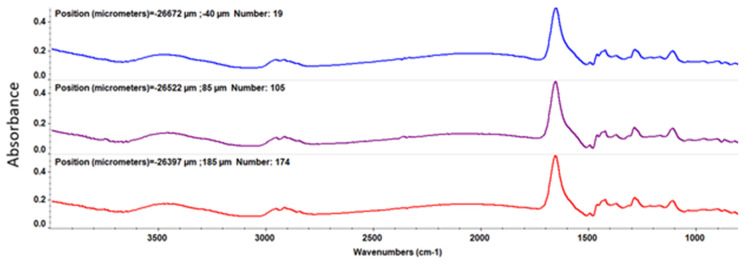
IR spectra of PVP/ZnO@LiN coatings at 200 mJ/cm^2^ laser fluence.

**Figure 8:**
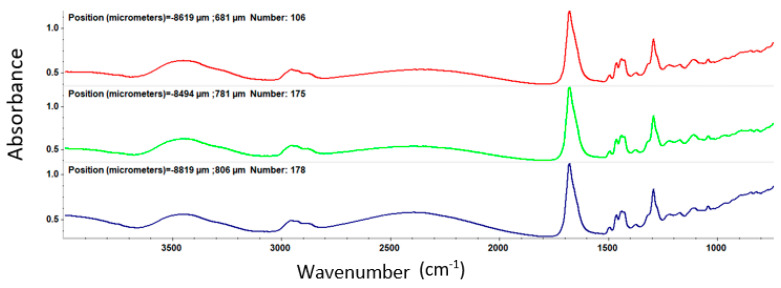
IR spectra of PVP/ZnO@LiN coatings at 300 mJ/cm^2^ laser fluence.

At a laser fluence of 200 mJ/cm^2^ (Figure 7), the intensity of absorption bands is low due to the reduced amount of sample deposited on the substrate, while, at a laser fluence of 300 mJ/cm^2^ (Figure 8), clear peaks are registered.

**Figure 9:**
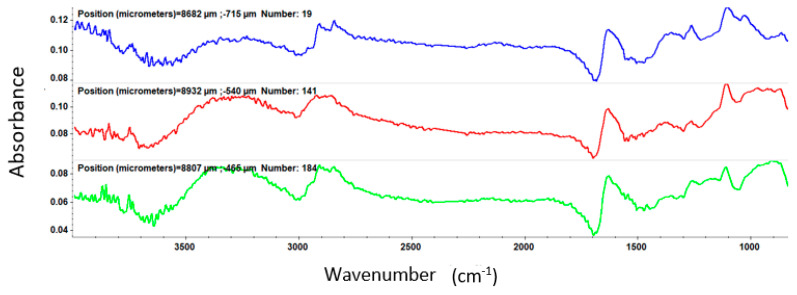
IR spectra of PVP/ZnO@LiN coatings at 400 mJ/cm^2^ laser fluence.

For the coatings deposited at 400 mJ/cm^2^ laser fluence (Figure 9), one can observe significant degradation of absorption bands intensities characteristic to the functional groups.

Through the IRM examination, an optimum sample deposition is found for a laser beam fluence of 300 mJ/cm^2^.

**Figure 10:**
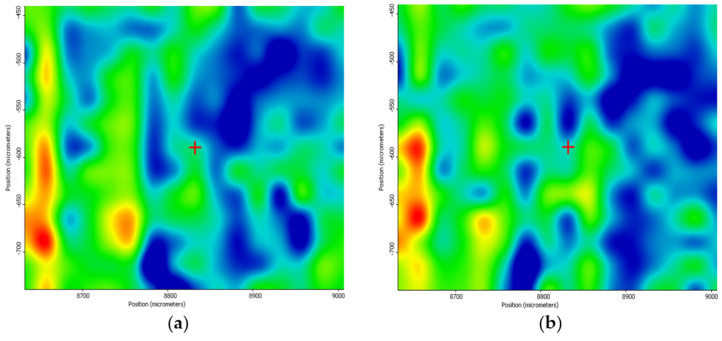
IR maps of PVP/ZnO@LiN dropcast based on the distribution of C–H (**a**) and C=O (**b**) bonds.

Supporting complementary information on the composition and chemical distribution of the coating layer was provided through the IR mapping of the samples. For identifying the optimum sample, the color distribution from the IR maps is considered as absorption intensity increases with the gradual change in color from blue to green, yellow, and red. It can be seen, in the case of the dropcast (Figure 10), that the predominant color is blue, indicating a minimum amount of sample on the substrate or even its absence.

**Figure 11:**
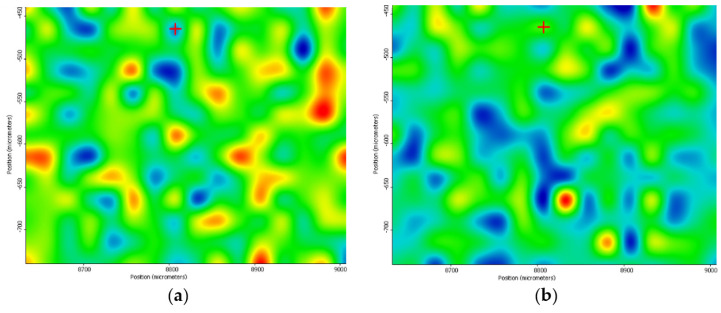
IR maps of PVP/ZnO@LiN based on the distribution of C–H (**a**) and C=O (**b**) bonds at 200 mJ/cm^2^ laser fluence.

In the case of the coatings prepared at 200 mJ/cm^2^ laser fluence, an improvement concerning the deposition on the silicon substrate can be observed (Figure 11). Although warmer colors are noticed, they are not uniformly distributed on the substrate, revealing that the transferred material is focused on small areas on the substrate surface.

**Figure 12:**
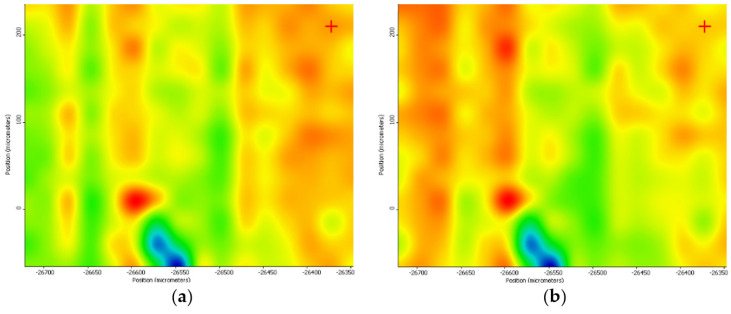
IR maps of PVP/ZnO@LiN based on the distribution of C–H (**a**) and C=O (**b**) bonds at 300 mJ/cm^2^ laser fluence.

Significant deposition enhancement is observed at a 300 mJ/cm^2^ laser fluence (Figure 12). In this case, the coatings are homogeneously deposited, there are no agglomerations or areas in which the sample material lacks.

**Figure 13:**
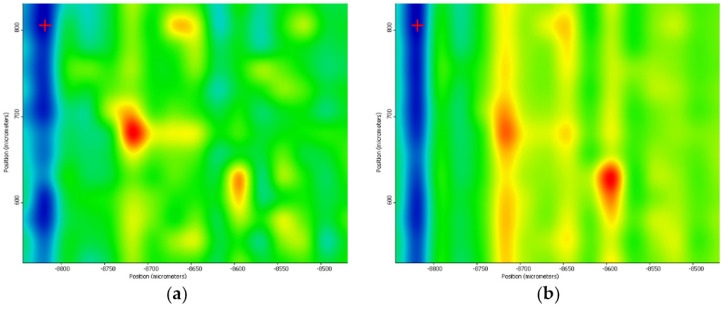
IR maps of PVP/ZnO@LiN based on the distribution of C–H (**a**) and C=O (**b**) bonds at 400 mJ/cm^2^ laser fluence.

The MAPLE deposition at 400 mJ/cm^2^ laser fluence resulted in the degradation of functional groups in the structure of the sample due to the high energy applied (Figure 13).

By analyzing the IR maps of these three experimental variants, it can be concluded that the most uniform chemical distribution and the lowest degree of functional group degradation were recorded in the case of 300 mJ/cm^2^ laser fluence. Therefore, the 300 mJ/cm^2^ laser fluence was selected as the optimal choice for MAPLE deposition of PVP/ZnO@LiN coatings regarding the compositional integrity and transfer efficiency. Considering the above discussed IR data, all coatings considered for further analyses were obtained using 300 mJ/cm^2^ laser fluence during MAPLE processing.

**Figure 14:**
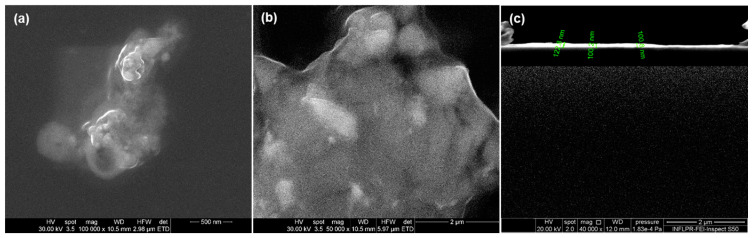
SEM micrographs of the thin coatings (**a**,**b**) and cross-section (**c**) prepared at 300 mJ/cm^2^ laser fluence.

The micrographs obtained for this experimental variant display a uniform coating deposited on the whole surface of the Si substrate. At a magnification of 100,000× (Figure 14a), NPs dimensions are between 25 and 30 nm, with irregular morphologies. Besides, the SEM image taken at 50,000× magnification (Figure 14b) shows the embedding of ZnO aggregates into the polymer matrix.

The cross-section analysis (40,000× magnification), Figure 14c, offers information on the thickness of the layer deposited. By means of data processing software, thickness measurements were performed, and we can observe that the coating dimensions vary between 100 and 125 nm.

### 3.3. Biological Evaluation of PVP/ZnO@LiN Coatings

#### 3.3.1. In Vivo Biocompatibility and Biodistribution of ZnO@LiN NPs

**Figure 15:**
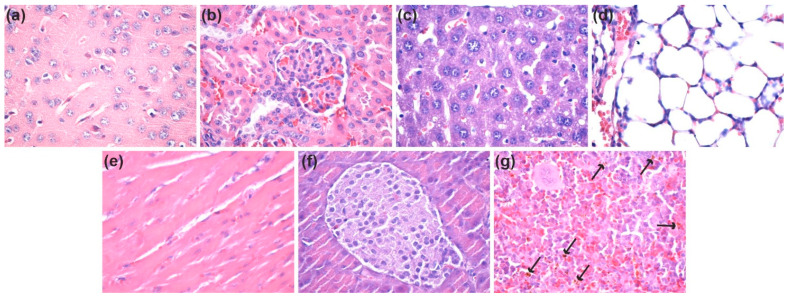
Micrographs of cross-sections through the mice internal organs treated with ZnO NPs after seven days (HE staining, ×400): (**a**) Brain; (**b**) Kidney; (**c**) Liver; (**d**) Lung; (**e**) Myocardium; (**f**) Pancreas; (**g**) Spleen (see arrows for ZnO@LiN NPs). HE: Hematoxylin–Eosin; NPs: Nanoparticles. (×100).

**Figure 16:**
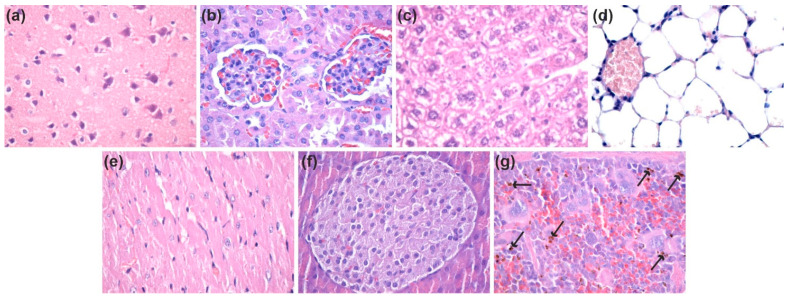
Micrographs of cross-sections through the mice internal organs treated with ZnO NPs after seven days (HE staining, ×400): (**a**) Brain; (**b**) Kidney; (**c**) Liver; (**d**) Lung; (**e**) Myocardium; (**f**) Pancreas; (**g**) Spleen (see arrows for ZnO@LiN NPs). HE: Hematoxylin–Eosin. (×100).

At 7 days and 14 days after the intraperitoneal administration of nanostructures, ZnO@LiN NPs were absent in the brain, kidney, liver, lung, myocardium, and pancreas (Figure 15a–f and Figure 16a–f). However, ZnO@LiN NPs were highlighted only in the red pulp of the spleen, in the cells of the macrophage system, both in the Billroth cords, and in the sinusoidal capillaries. At 14 days, ZnO@LiN NPs exhibited higher amounts than those recorded in samples taken after 7 days. The ZnO@LiN NPs appeared as brownish-black, granular, agglomerated, spherical structures of variable dimensions, with up to 3 µm diameter. The density of the ZnO@LiN NPs varied depending on cell type, some red pulp cells exhibiting a larger number of endocytosed NPs (Figure 15g and Figure 16g). Hypertrophy of the white pulp was observed because the engulfment of ZnO@LiN NPs stimulated the formation of macrophages with multilobular nuclei.

#### 3.3.2. Antibacterial Efficiency

**Figure 17:**
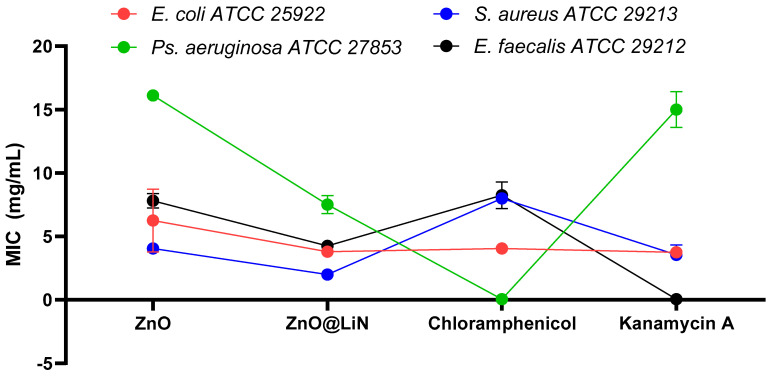
Comparison of MIC values of ZnO-based nanoparticles and genera use antibiotics against strains of *E. coli*, *P. aeruginosa*, *S. aureus*, and *E. faecalis*.

In order to quantify the antimicrobial effect of the prepared nanoparticles, a minimum inhibitory concentration (MIC) assay was performed. The antibacterial efficiency of ZnO@LiN NPs was compared with that of bare ZnO NPs and broad-spectrum antimicrobial agents (i.e., chloramphenicol and kanamycin A, utilized as recommended by CLSI 2019) [48] commonly used in the treatment of severe infections. (Figure 17). The obtained results demonstrate the strongest antimicrobial activity for ZnO@LiN NPs against all tested pathogens. These NPs are observed to be especially efficient against *S. aureus* strain, its MIC value being much lower than the values obtained for chloramphenicol and kanamycin A. Nonetheless, its antimicrobial behavior against *P. aeruginosa* is significantly stronger than that of kanamycin A.

#### 3.3.3. Microbial Biofilm Modulation

**Figure 18:**
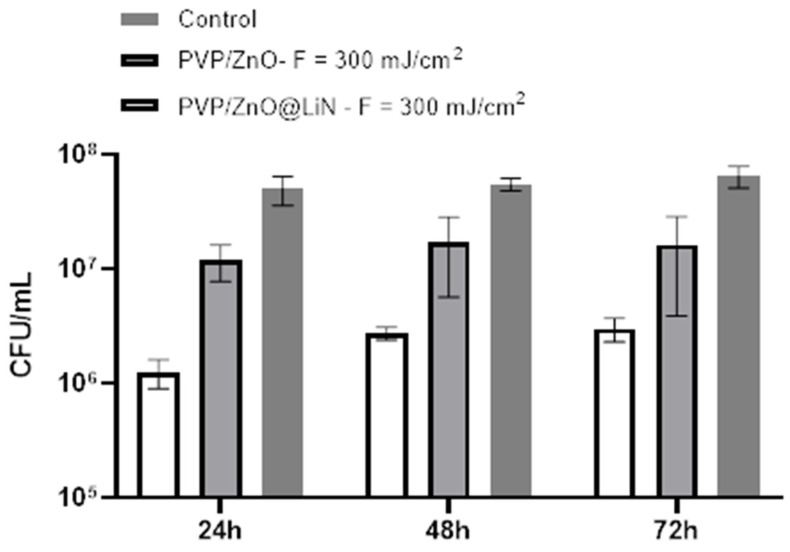
Evaluation of microbial biofilm development at 24, 48, and 72 h after incubation with *E. coli* in the presence and absence of PVP/ZnO@LiN coatings.

Next, we have investigated the antibiofilm properties of the developed coatings based on ZnO@LiN NPs. The substrates coated with PVP/ZnO@LiN thin films were subjected to in vitro tests utilizing a static monospecific biofilm model. The antimicrobial results (Figure 18) demonstrate that the linalool-functionalized coatings diminishes the biofilm formation degree and reduces bacterial adhesion, being more efficient than the uncoated control sample and ZnO NPs samples. The highest inhibitory effect is noticed in the first 24 h, slightly diminishing after 48 and 72 h, respectively. In contrast, control substrates present high amounts of CFU/mL, indicating a pronounced tendency to be colonized with *E. coli*. ZnO NPs samples express a moderate anti-biofilm activity inhibitor as compared to coatings control samples, but less efficient as compared to ZnO@LiN NPs coatings.

**Figure 19:**
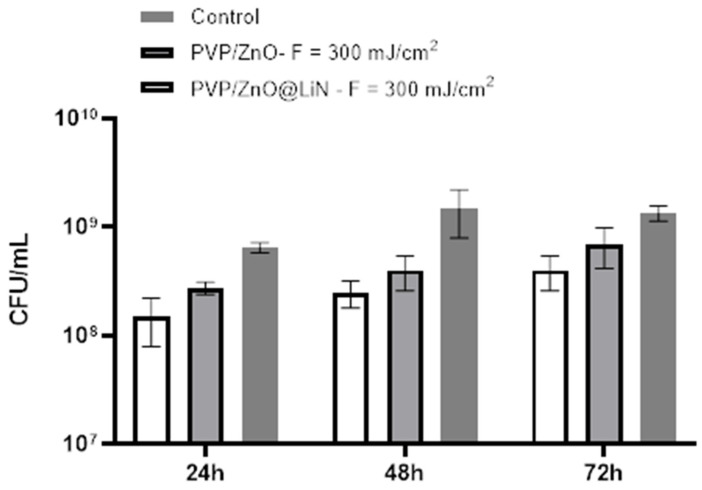
Evaluation of microbial biofilm development 24, 48, and 72 h after incubation with *P. aeruginosa* in the presence and absence of PVP/ZnO@LiN thin coatings.

The anti-adherent potential of PVP/ZnO@LiN against *P. aeruginosa* is lowered as compared to *E. coli* biofilm development. A stronger activity against *P. aeruginosa* is observed in the first 24 h, while anti-adherent properties decrease after 48 and 72 h (Figure 19).

**Figure 20:**
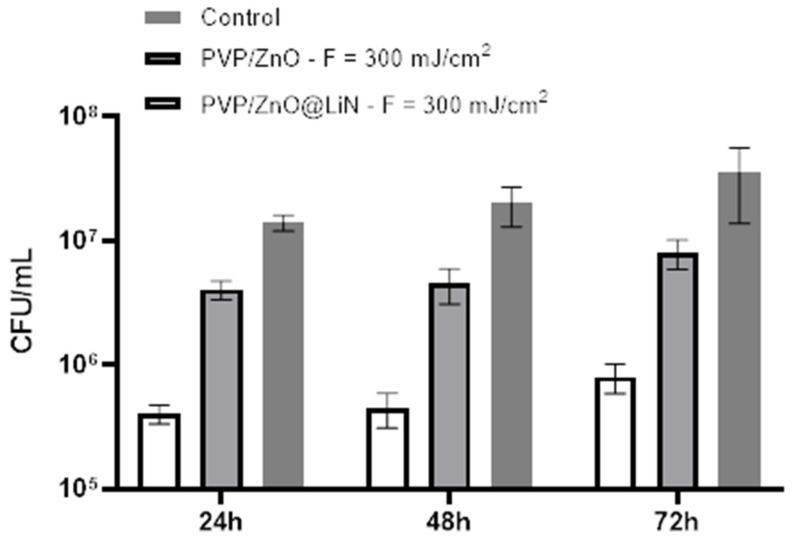
Evaluation of microbial biofilm development at 24, 48, and 72 h after incubation with *S. aureus* in the presence and absence of PVP/ZnO@LiN coatings.

In what concerns the effect of PVP/ZnO@LiN against *S. aureus*, similar anti-adherent properties are observed throughout the entire incubation period (Figure 20). Moreover, although the mature biofilm is more resistant to antimicrobial agents, the CFU/mL values associated with functionalized substrates remain constant at the different tested points in time, suggesting the constant antimicrobial efficiency of the obtained nanostructured coatings for at least three days.

**Figure 21:**
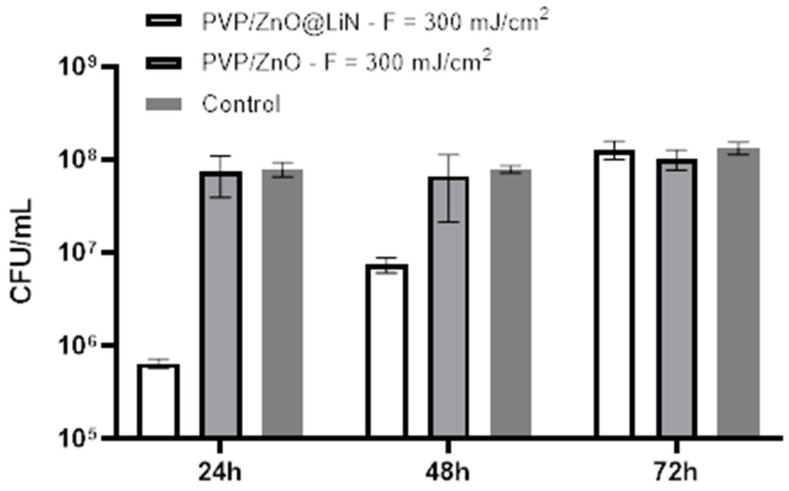
Evaluation of microbial biofilm development at 24, 48, and 72 h after incubation with *E. faecalis* in the presence and absence of PVP/ZnO@LiN coatings.

The antimicrobial test of PVP/ZnO@LiN performed against *E. faecalis* shows the best result in the 24 h compared to the control sample (Figure 21). The efficiency of the composite material drastically reduces in time. After 72 h, the same effect is observed as for the control sample, reflecting the proliferation of the bacterial strain in similar ways on both control and nanocoated material.

## 4. Conclusions

This study presented the successful synthesis of ZnO NPs, their functionalization with linalool, and subsequent MAPLE deposition to obtain coatings with bacterial inhibitory activity. An optimum deposition was observed for a laser fluence of 300 mJ/cm^2^. A potent antibacterial activity was registered for the ZnO@LiN samples, their inhibitory effects on biofilm formation being stronger than ZnO and some broad-spectrum antibiotics. Through in vitro assays on prokaryote cells, it was proven that ZnO@LiN hinders microbial biofilm formation of both Gram-positive and Gram-negative bacteria strains. Thus, the obtained results recommend these nanostructured coatings as candidates for developing new surfaces or biomedical devices with low costs and high efficiency, representing a promising strategy in preventing biofilm infections.

## Data Availability

The data presented in this study are available on request from the corresponding author.
